# Feasibility of continuous glucose monitoring in an acute geriatric unit – the GLYCOGER study

**DOI:** 10.1186/s12877-026-07333-w

**Published:** 2026-03-17

**Authors:** Nathanaël Bassas Letissier, Helena Mosbah, Pierre-Jean Saulnier, Xavier Piguel, Marc Paccalin, Evelyne Liuu

**Affiliations:** 1 Geriatric Department, CHU Poitiers, Poitiers, France; 2 Department of Endocrinology, Diabetology & Nutrition, CHU Poitiers, Poitiers, France; 3 Clinical Investigation Centre, CIC 1402, CHU Poitiers University of Poitiers, INSERM, Poitiers, France

**Keywords:** Diabetes mellitus, Aged, Geriatrics, Continuous Glucose Monitoring

## Abstract

**Background:**

Continuous glucose monitoring (CGM) enhances glycemic control and reduces hypoglycemia in patients with diabetes, but its use in geriatric inpatients remains limited.

**Aim:**

To assess the feasibility in CGM use among patients hospitalized in an acute geriatric unit.

**Methods:**

We conducted a pilot single-center observational study involving patients aged ≥ 75 years with insulin-treated diabetes who met the French national criteria for CGM reimbursement. The primary endpoint was feasibility of CGM, defined as the acceptability and continuation of CGM during hospitalization with ≥ 70% sensor activity. Secondary endpoints included hypoglycemia (< 70 mg/dL), factors associated with hypoglycemia and hyperglycemia (> 180 mg/dL), and the clinical, metabolic, and therapeutic determinants of CGM results.

**Results:**

Among 52 patients (median age 85.5 years; 50% women), 46 (89%) continued CGM during hospitalization and 40 (77%) achieved ≥ 70% sensor activity. A total of 61 hypoglycemic episodes were recorded (mean 1.2 per patient), none of which were symptomatic. Mean time below range (< 70 mg/dL) was 1.4 ± 2.1%, and mean time above range (> 180 mg/dL) was 31.9 ± 22.4%. Exploratory analyses showed that time above range was positively correlated with bolus (ρ = 0.72, *p* < 0.001) and total daily insulin doses (ρ = 0.68, *p* < 0.001). At discharge, 38 of the 41 patients (96.7%) who remained on insulin were prescribed CGM. No device-related serious adverse events were observed.

**Conclusions:**

CGM use in an acute geriatric unit was feasible and well tolerated, with a high proportion of analyzable sensor data and limited hypoglycemia. These findings support the integration of CGM into routine inpatient geriatric diabetes care.

**Supplementary Information:**

The online version contains supplementary material available at 10.1186/s12877-026-07333-w.

## Introduction

Diabetes is a prevalent condition that increases with age, predominantly driven by type 2 diabetes in older adults. In 2021, there were 537 million people with diabetes worlwide [1] and in 2024, age-standardized prevalence of diabetes was 6.5% in France [2]. One in five men aged between 70 and 85 and one in seven women aged between 75 and 85 have medically treated diabetes [3]. When a patient with diabetes is hospitalized in acute care, implementation of a multiple daily insulin injection protocol involving iterative capillary blood glucose monitoring may be necessary to mitigate blood sugar imbalance [4]. Fear of hypoglycemia, which is highly prevalent and potentially severe in this population, frequently affects therapeutic decision-making [5–7]. Since 2017, an innovative alternative with continuous glucose monitoring (CGM) systems has become widely available, and since 2023, French health authorities have allowed any medical practitioner to initiate CGM contingent upon patient education, with re-evaluation of its benefits after three months of monitoring [8, 9]. Developed for the purpose of providing real-time monitoring of interstitial glucose, CGM measures the concentration of glucose in the interstitial fluid [10] and correlates well with blood glucose meters [11]. The most recent studies demonstrate that CGM enhances diabetes management, particularly among individuals over the age of 65, with a 34% reduction in hospitalizations over one year after its introduction [12]. In France, the main CGM systems currently available for patients with type 2 diabetes are the FreeStyle Libre 2^®^ (Abbott) and the Dexcom One+^®^ (Dexcom). Reimbursement by the French national health insurance system is granted for patients treated with multiple daily insulin injections or continuous subcutaneous insulin infusion, and more recently for selected patients on basal insulin therapy with insufficient glycemic control, according to national reimbursement criteria. CGM is now routine care in diabetes units. However, despite its proven benefits, CGM is still not widely used in geriatric wards in France [13]. The aim of this study was to assess the feasibility of CGM use in patients aged over 75 years hospitalized in an acute geriatric unit.

## Methods

### Study design

The GLYCOGER study is a single-center observational feasibility study conducted in an acute geriatric unit at Poitiers University Hospital, France. All healthcare professionals received standardized training, provided by a clinical nurse trained in therapeutic education, in the use of CGM. After enrollment, patients were equipped with an intermittently scanned continuous glucose monitoring system. The CGM system used in the study was the FreeStyle Libre 2^®^ (isCGM; FreeStyle Libre 2; Abbott Diabetes Care, Witney, United Kingdom). The sensor was applied to the posterior aspect of the upper arm in accordance with the manufacturer’s instructions. Glucose values were obtained using the dedicated reader device provided by the manufacturer. No smartphone application was used, as smartphones were not available in our hospitalized geriatric population. Scans were performed by nurses and patients after receiving in-hospital training on device use. Both patients and healthcare staff had unrestricted access to all real-time CGM data. The glycemic target range was set at 70–180 mg/dL, with hypoglycemia alarms programmed at ≤ 80 mg/dL to minimize the time spent in hypoglycemia (defined as glucose values below 70 mg/dL). These thresholds were chosen in accordance with international recommendations [14], which recommend that time spent in hypoglycemia be < 1%. Insulin regimens and other antidiabetic treatments were prescribed at the discretion of the attending physicians. No predefined protocol required systematic confirmation of CGM-detected hypoglycemic values by capillary glucose testing. Capillary measurements were performed according to routine clinical practice and at the discretion of the medical team in case of suspected discrepancy or clinical symptoms.

### Selection criteria

Patients were recruited consecutively between February 1st and December 31st, 2024. The inclusion criteria were: (1) age ≥ 75 years, (2) history of diabetes requiring insulin therapy, (3) type 1 diabetes, or type 2 diabetes, secondary diabetes, or iatrogenic diabetes, and (4) at least one of the criteria required for CGM reimbursement in France: (i) a multiple daily injection regimen, (ii) a basal insulin regimen with HbA1c > 8% (> 10.6 mmol/L) [15].

Exclusion criteria were: (1) refusal or inability to provide informed consent (due to cognitive impairment or delirium), and (2) admission for palliative care with a life expectancy < 3 months.

Although type 2 diabetes was predominant in the cohort, patients with other forms of insulin-treated diabetes were not excluded if they met CGM indication criteria.

### The variables studied

Baseline demographic, clinical, functional, therapeutic, and biological characteristics were collected at admission. Medication use was documented by the number of treatments per day at admission and discharge, classified into three categories: no polypharmacy (< 5 drugs/day), moderate polypharmacy (5–9 drugs/day), and severe polypharmacy (≥ 10 drugs/day). Biological parameters collected at admission included fasting glucose, glycated hemoglobin, creatinine, and estimated glomerular filtration rate (eGFR). HbA1c was measured on EDTA-anticoagulated whole blood using an immunoturbidimetric inhibition assay (TINIA) by absorption spectrophotometry on a Roche cobas c503 analyzer (Roche Diagnostics).

Insulin treatment during hospitalization was characterized by mean daily basal insulin dose, mean daily bolus dose, mean number of prandial boluses, and total daily dose, recorded from inclusion until discharge. Regimens were further categorized according to the average daily number of rapid-acting insulin boluses: patients receiving < 1 bolus/day were on basal insulin alone; those receiving 1–2 boluses/day were classified as receiving basal insulin with rapid-acting correction doses; and those receiving > 2 boluses/day were defined as being on a basal–bolus regimen.

At discharge, data from CGM were summarized, including percentage of data capture, number of hypoglycemic events (defined by glycemic records below 70 mg/dL, as no episode, one, 2–4, or ≥ 5), glycemic variability, and mean glucose level and glycemic targets. Glycemic targets were defined according to international consensus recommendations for CGM, including those of the ADA [16] and the international consensus [14]. Targets were defined as time in range (TIR) 70–180 mg/dL, time below range (TBR) < 70 mg/dL with a recommended threshold < 1% for older adults, and time above range (TAR) > 180 mg/dL. In addition to TIR, TBR, and TAR, time in tight range (TITR) was calculated, defined as the percentage of time spent between 70 and 140 mg/dL, in patients with ≥ 70% sensor activity. Adverse events related to CGM, such as skin reactions, were also recorded.

### Endpoints

The primary endpoint was the feasibility of CGM in geriatric care evaluated through: (i) the proportion of patients who consented to participate in the study and continued CGM throughout hospitalization, (ii). the proprotion of patients achieving ≥ 70% sensor activity, in accordance with international recommendations [14], (iii) continuation of CGM at discharge, defined as a CGM prescription in patients remaining eligible for insulin therapy.

Secondary endpoints included: number of hypoglycemic episodes (< 70 mg/dL), TBR, TAR, TIR, and TITR, and clinically relevant glycemic metrics for older patients. Clinical, biological, and therapeutic factors associated with CGM use were also assessed.

### Statistical analysis

Baseline characteristics were summarized according to the type and distribution of the variables. Quantitative variables were expressed as mean ± standard deviation (SD) when normally distributed, or as median and interquartile range (IQR) otherwise. Normality of continuous variables was assessed using visual inspection (histograms and Q–Q plots) and the Shapiro–Wilk test. Non-parametric tests were used when appropriate. Time-in-range metrics (TIR, TBR, TAR, and TITR) were expressed as mean ± SD in accordance with international reporting standards, although distributions were assessed for normality and non-parametric tests were applied when appropriate. Count variables, such as hypoglycemic events, were described using means and ranges.

For CGM-derived analyses, only patients with at least 7 consecutive days of CGM recording were included, in accordance with international recommendations for CGM data interpretation. Patients were categorized post hoc according to sensor activity rate (< 70% vs. ≥ 70%) for analytical purposes. The ≥ 70% threshold was selected in accordance with international recommendations for CGM data interpretation to ensure reliable calculation of glycemic metrics [17].

CGM-derived glycemic metrics (TIR, TBR, TAR, TITR, and hypoglycemic events) were calculated only for patients with at least 7 days of CGM recording and a sensor activity ≥ 70%.

Group comparisons were performed using Student’s t-test or Mann–Whitney U test, as appropriate. Qualitative variables were described as counts and percentages, with group comparisons using Chi-squared test or Fisher’s exact test. The relationship between mean daily insulin doses (basal, bolus, and total), calculated over the CGM observation period, and glycemic targets was analyzed using Spearman correlation analysis. A two-sided p-value ≤ 0.05 was considered statistically significant. Analyses were carried out using R version 4.5.2 (R Foundation for Statistical Computing, Vienna, Austria). Initial exploratory analyses were performed using XLStat^®^ (Addinsoft, Paris, France).

### Ethics and consent

Oral informed consent was obtained from all participants after the provision of written study information, in accordance with national regulations for observational studies based on routine care. The study complied with the principles of Good Clinical Practice and was registered by the French data protection authority (Commission Nationale de l’Informatique et des Libertés, CNIL; registration number 24501189).

## Results

### Characteristics of patients

Between February and December 2024, 57 consecutive patients were pre-screened, and 52 were finally included (91%). Five patients (9%) declined participation; in all cases, refusal was related to fear of the new technological device and apprehension about its use.

The cohort consisted of 26 women (50%) and 26 men (50%), with a median age of 85.5 years (IQR: 80.0–88.3), a median BMI of 28.9 kg/m² (IQR: 23.2–34.5). The median CIRS-G score was 13.0 (IQR: 10.8–16.0) (Table 1). Most patients lived at home prior to admission (*n* = 47, 90%). The leading causes of admission to the acute geriatric unit were cardiovascular diseases (*n* = 15, 29%) and falls (*n* = 12, 23%). Almost all patients (*n* = 50, 96%) had type 2 diabetes, while two cases were classified as iatrogenic diabetes (one secondary to immunotherapy and one to glucocorticoids). Baseline characteristics were largely similar between patients with sensor activity ≥ 70% and < 70%, with no statistically significant differences (Supplementary Table 1).

**Table 1:** Clinical and demographic characteristics

	Total (*n* = 52, 100%) Median (IQR) or *n* (%)
Patients’ characteristics	
Age, years	85.5 (80.0-88.3)
Gender - Female	26 (50.0)
BMI (kg/m²)	28.9 (23.2–34.5)
CIRS-G	13.0 (10.8–16.0)
ADL (0–6)	5.0 (3.5-6.0)
iADL (0–8)	3.5 (1.0–5.0)
Living situation prior to admission	
Home	47 (90.4)
Nursing home	4 (7.7)
Residential care facility	1 (1.9)
Number of drugs per day at admission	10.0 (8.0–12.0)
Biological values at admission	
Fasting glucose (mg/dL)	160.0 (110.0-210.0)
Glycated hemoglobin (mmol/L)	10.1 (8.9–11.9)
Glycated hemoglobin (%)	8.0 (7.3–8.7)
Creatinine (µmol/L)	101.5 (77.0-147.0)
eGFR (mL/min/1,73 m²)	50.0 (36.0-71.5)
History of diabetes	
Type of diabetes	
Type 2 diabetes	50 (96.2)
Secondary	2 (3.8)
Diabetes Duration (years)	
< 10	19 (36.5)
≥ 10	33 (63.5)
Antidiabetic drugs prior to hospital admission	
Oral antidiabetic only	17 (32.7)
Insulin only	16 (30.8)
Oral antidiabetic and insulin	16 (30.8)
None	3 (5.8)
Diabetes complications	
Any microangiopathic complication	41 (78.9)
Retinopathy	8 (15.4)
Nephropathy*	33 (63.5)
Neuropathy	11 (21.2)
Any macroangiopathic complication	36 (69.2)
Stroke	19 (36.5)
Myocardial infarction	17 (32.7)
Peripheral arterial disease	11 (21.2)

*BMI* Body mass index, *CIRS-G* Cumulative illness rate scale geriatric, *ADL* Activities of daily living, *iADL* Instrumental activities of daily living, *eGFR* Estimated glomerular filtration rate

Data are presented as median (IQR) or n (%)

*Defined as eGFR ≤ 60 mL/min/1.73 m² according to MDRD

### Antidiabetic treatments

Before hospitalization, 17 (33%) patients were receiving oral antidiabetic drugs (OADs) alone, 16 (31%) insulin alone, 16 (31%) a combination of insulin and OADs, while 3 (6%) patients were not receiving any antidiabetic treatment. According to French reimbursement criteria, 32 (62%) patients already met eligibility requirements for CGM prior to admission (basal insulin with HbA1c > 8%), yet none were using CGM at baseline. During hospitalization, 25 (48%) patients received a basal–bolus regimen (> 2 boluses/day on average) with a mean daily insulin dose of 0.51 ± 0.14 IU/kg; 14 (27%) a basal regimen with rapid-acting insulin correction doses (1–2 boluses/day) at 0.29 ± 0.12 IU/kg; and 13 (25%) basal insulin only (< 1 bolus/day) at 0.15 ± 0.08 IU/kg. Details of basal and bolus insulin doses during the hospital stay are provided in Supplementary Fig. 1. At discharge, 6 (12%) patients were on OADs alone, 19 (37%) on insulin alone, and 23 (44%) on a combination of insulin and OADs.

### Primary endpoint: feasibility of CGM use in an acute geriatric unit

The primary endpoint of the study was the feasibility of CGM use during hospitalization in an acute geriatric unit. Among the 52 included patients, 46 (89%) continued CGM throughout their hospital stay. In addition, 40 (77%) patients achieved a sensor activity rate ≥ 70%, meeting the international recommendations for reliable CGM data interpretation. CGM was initiated early during hospitalization and used for a median duration of 14 days (IQR: 10–24), corresponding to a substantial proportion of the hospital stay.

**Fig. 1:**
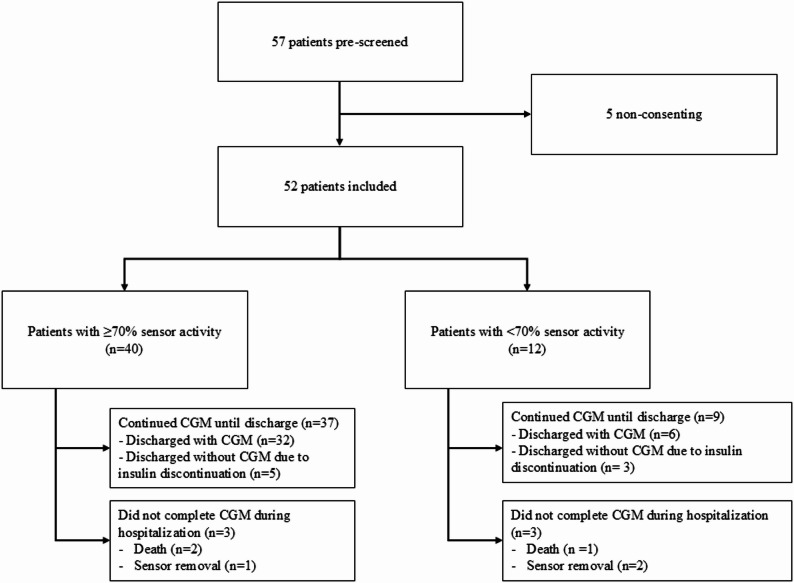
Flow chart of the study. Between February and December 2024, 57 patients meeting inclusion criteria were approached. Five patients declined participation; the sole reason for refusal was fear of using a new technological device. CGM-derived glycemic analyses were performed exclusively in patients with ≥ 70% sensor activity (*n* = 40)

Discontinuation of CGM was infrequent and primarily related to clinical circumstances rather than device intolerance, including in-hospital death (*n* = 3) or repeated sensor removal (*n* = 3). At discharge, 38 of the 41 patients who remained on insulin therapy were prescribed CGM. Three patients did not receive a CGM prescription at discharge due to sensor removal during hospitalization. Overall, these findings demonstrate high feasibility and acceptability of CGM use in an acute geriatric setting (Fig. 1).

When comparing patients according to the 70%-sensor activity threshold, no statistically significant differences were observed in demographic characteristics, diabetes duration, or vascular complications (Supplementary Table 1).

### Glycemic metrics

**Fig. 2:**
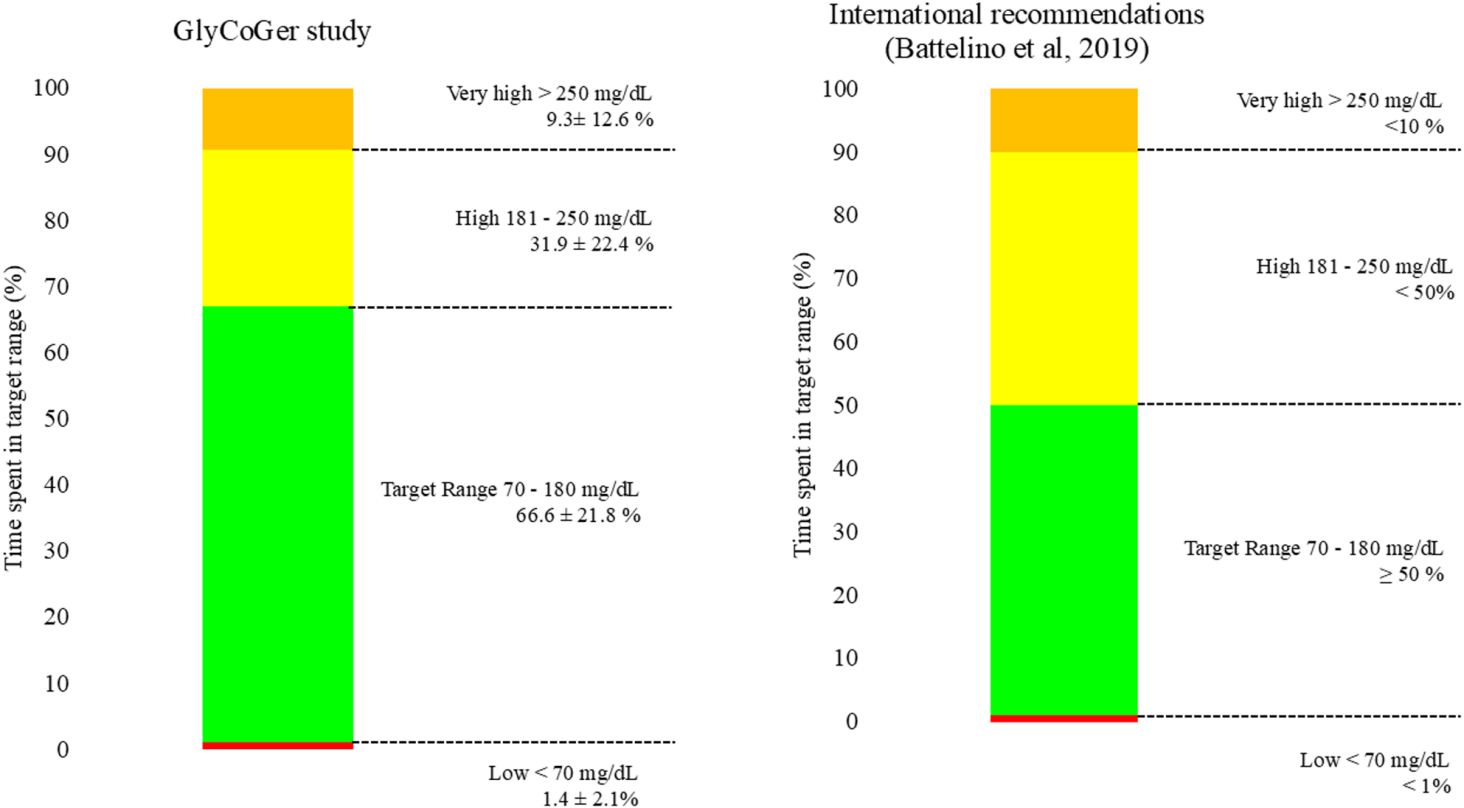
Continuous glucose monitoring report synthesis. Distribution of time spent in glycemic ranges derived from CGM data in patients with ≥ 70% sensor activity (*n* = 40), compared with international CGM consensus targets [14]

Among patients with ≥ 70% sensor activity, TBR < 70 mg/dL was 1.4 ± 2.1%, and the mean time above range (TAR > 180 mg/dL) was 31.9 ± 22.4% (Table 2). The median coefficient of variation was 30.4% (IQR: 24.1–33.7). A detailed overview of glycemic profiles and their comparison with international recommendations is provided in Fig. 2.

**Table 2:** CGM-derived glycemic metrics in patients with ≥ 70% sensor activity (*n* = 40)

	Mean ± SD	Median (IQR)	Range
TIR 70–180 mg/dL (%)	66.6 ± 21.8	71 (51–86)	13–100
TBR < 70 mg/dL (%)	1.4 ± 2.1	0 (0–3)	0–7
TBR < 54 mg/dL (%)	0 ± 0.2	0 (0–0)	0–1
TAR > 180 mg/dL (%)	31.9 ± 22.4	28 (10.8–48.8)	0–87
TAR > 250 mg/dL (%)	9.3 ± 12.6	4 (0-12.5)	0–52
Coefficient of variation (%)	30.2 ± 7.8	30.4 (24.1–33.7)	15.5–51.5
Mean glucose (mg/dL)	161.8 ± 35.4	154 (130.2–188)	110–253
TITR (%)*	41.8 ± 23.1	49 (21-55.5)	0–91

Data are presented as mean ± Standard Deviation (SD), median (Interquartile Range, IQR), and range. Normality of continuous variables was assessed using the Shapiro-Wilk test

*TIR* Time in Range, percentage of time with sensor glucose between 70 and 180 mg/dL. *TBR* Time Below Range, percentage of time with sensor glucose below 70 mg/dL (TBR < 70 mg/dL) or below 54 mg/dL (TBR < 54 mg/dL). *TAR* Time Above Range, percentage of time with sensor glucose above 180 mg/dL (TAR > 180 mg/dL) or above 250 mg/dL (TAR > 250 mg/dL). TAR > 180 mg/dL includes both TAR 180–250 mg/dL and TAR > 250 mg/dL. *TITR* Time in Tight Range, percentage of time with sensor glucose between 70 and 140 mg/dL. *CV* Coefficient of Variation, a measure of glycemic variability, calculated as the ratio of the standard deviation to the mean glucose level

Glycemic targets are defined according to the international consensus on CGM metrics [14]: TIR > 50%, TBR < 1%, TAR < 50% for older adults with insulin-treated diabetes

* TITR was available in 39 of 40 patients (one missing value)

### Hypoglycemia

Hypoglycemic events were recorded across the entire cohort (*n* = 52), as CGM alarms were active in all patients regardless of sensor activity rate, enabling reliable detection of hypoglycemic episodes independently of the ≥ 70% threshold applied for time-in-range metrics. A total of 61 hypoglycemic episodes were recorded, corresponding to a mean of 1.2 episodes per patient (range: 0–8). Half of the patients did not experience any hypoglycemic episodes. All hypoglycemic events were asymptomatic. No association was observed between the occurrence of hypoglycemia and age, sex, functional status, comorbidity burden, diabetes duration, or diabetes-related complications. Detailed comparisons are provided in Supplementary Table 2.

Among the 40 patients with ≥ 70% sensor activity, 26 (65%) experienced at least one hypoglycemic episode. No significant difference in weight-adjusted insulin doses was observed between patients with and without hypoglycemia (total insulin: 0.35 vs. 0.40 IU/kg/day, *p* = 0.372; basal insulin: 0.20 vs. 0.24 IU/kg/day, *p* = 0.301; bolus insulin: 0.14 vs. 0.18 IU/kg/day, *p* = 0.681).

### Hyperglycemia

Among patients who did not meet the TAR target (> 50% above 180 mg/dL, *n* = 15, 37.5%), weight-adjusted total daily insulin doses were observed to be higher compared to those meeting the target (0.50 vs. 0.29 IU/kg/day, *p* < 0.001). Similar differences were observed for basal insulin (0.25 vs. 0.18 IU/kg/day, *p* = 0.009) and bolus insulin (0.25 vs. 0.11 IU/kg/day, *p* < 0.001).

### Exploratory correlations between insulin doses and CGM-derived metrics

Exploratory analyses were performed to assess the relationship between insulin dosing and CGM-derived glycemic metrics among patients with ≥ 70% sensor activity. Higher total daily insulin doses were positively correlated with greater hyperglycemic exposure, as reflected by higher time above range (TAR), and negatively correlated with time in range (TIR). Similar patterns were observed for bolus insulin doses, whereas basal insulin doses showed weaker correlations.

**Fig. 3:**
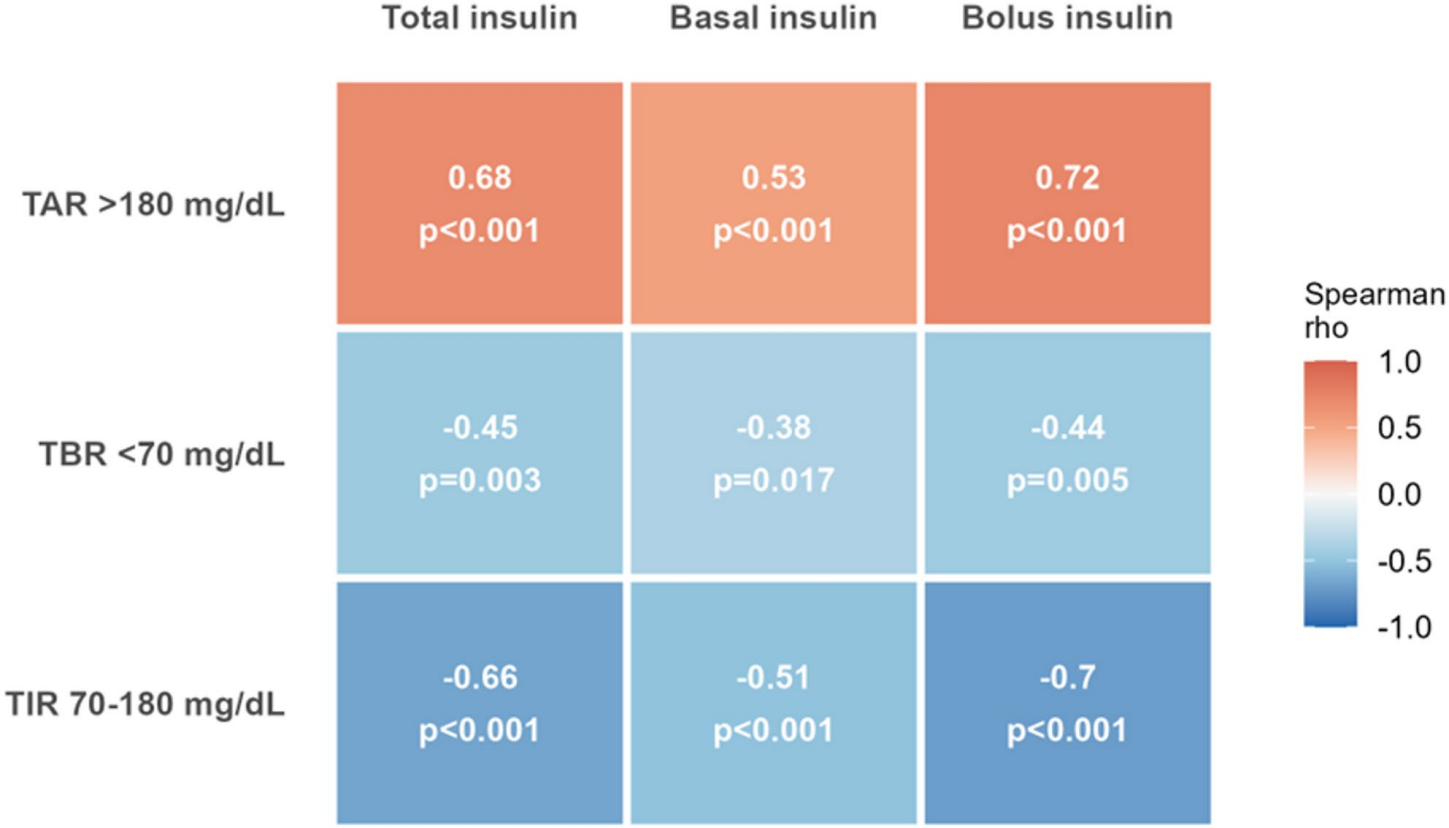
Spearman correlations between mean daily insulin doses (total, basal, and bolus) and CGM-derived glycemic metrics in patients with ≥ 70% sensor activity (*n* = 40). Color intensity reflects the strength of the Spearman correlation coefficient (rho): blue indicates negative correlations, red indicates positive correlations. Values within each cell represent the Spearman rho coefficient and the corresponding two-sided p-value. TIR, Time in Range: percentage of time with sensor glucose between 70 and 180 mg/dL. TAR, Time Above Range: percentage of time with sensor glucose above 180 mg/dL (TAR 180–250 mg/dL and TAR > 250 mg/dL combined). TBR, Time Below Range: percentage of time with sensor glucose below 70 mg/dL. IU, international units. Analysis restricted to patients with ≥ 70% sensor activity (Group 1, *n* = 40)

These associations are summarized in Fig. 3, which presents a heatmap of Spearman correlation coefficients between insulin doses and CGM metrics. Given the exploratory nature of these analyses and the study design, no causal inference can be drawn.

## Discussion

This pilot study demonstrates the high feasibility of CGM in older inpatients with multiple long-term conditions, with analyzable glycemic curves during hospitalization reaching 77%. To our knowledge, it is one of the first studies to evaluate CGM during acute geriatric hospitalization.

Although patient-reported outcomes were not formally assessed, CGM has been shown in previous studies to reduce the burden associated with frequent capillary blood glucose testing. A meta-analysis comparing CGM with self-monitoring of blood glucose in patients with type 1 and type 2 diabetes reported improved treatment satisfaction, and reduced discomfort associated with repeated fingerstick measurements [18]. By limiting the need for iterative capillary testing, CGM may reduce procedure-related discomfort and improve practicality of glucose monitoring. This aspect may be particularly relevant in older adults with impaired vision, reduced manual dexterity, or functional limitations that complicate traditional fingerstick-based monitoring [16, 19]. In our study, no skin-related adverse events were observed during CGM use. Although our sample size and duration of CGM exposure do not allow firm conclusions regarding safety, this finding is consistent with previous studies reporting a low incidence of cutaneous adverse events associated with CGM use in older adults [20].

The continuation of CGM in 88% of patients suggests its practical relevance in the management of diabetes in acute situations in older people. This finding is consistent with the study by Leite et al. In an insulin-treated adult population aged over 65, with total follow-up of six weeks at home, the result showed high adherence to CGM (over 91%) [21]. Our findings are consistent with recent reviews highlighting the feasibility and clinical utility of CGM in acute care settings, while underlining the practical barriers that still limit its widespread adoption in hospital practice [22].

The factors contributing to optimal adherence to CGM are multifaceted, encompassing both patient-reported satisfaction (comfort, reduced burden of fingerstick) and the acceptance and preparedness of healthcare teams. Patient-reported outcomes and qualitative studies show that treatment satisfaction, quality of life, and self-efficacy strongly influence CGM uptake and persistence [23].

Consequently, a structured pre-implementation phase including dedicated training for healthcare professionals is considered essential prior to study initiation; implementation literature and nursing reports emphasize that staff education, clear and standardized protocols, and local champions are critical to safe and effective CGM deployment in hospital settings [24].

Interestingly, in our cohort, younger age, functional independence (assessed by ADL), and duration of diabetes were not associated with higher sensor activity. While factors such as age, functional status, or comorbidity burden are often considered determinants of technology adherence in older adults, previous studies suggest that these individual characteristics alone do not fully explain CGM use and data completeness. Organizational factors, support from healthcare professionals, and contextual elements also play a substantial role [25]. Socioeconomic or educational status has been shown to influence self-management capacities and technology adoption in diabetes care [26]. Moreover, data completeness is unlikely to rely primarily on patient-related factors, but rather in procedural aspects such as insufficient scanning by either the patient or the healthcare staff during hospitalization. This underscores the importance of staff engagement when introducing new technologies in geriatric care settings.

Several practical barriers to CGM implementation in acute geriatric settings should be acknowledged. In our study, fear of new technology was the main reason for refusal among eligible patients, highlighting patient-related barriers. Limited access to smartphones also influenced feasibility, as CGM data were accessed exclusively via readers. From an organizational perspective, variability in scan frequency and the absence of standardized protocols for CGM interpretation may affect data completeness. In addition, despite clear reimbursement criteria, CGM was not routinely prescribed prior to hospitalization, reflecting persistent barriers at the interface between hospital and outpatient care. These findings underscore the need for structured implementation strategies to optimize CGM use in geriatric populations [25].

Hospitalization in an acute geriatric unit may represent a particularly suitable setting for CGM initiation in older adults. During the hospital stay, patients benefit from close medical and nursing supervision, allowing secure device placement, monitoring of alarms, and timely interpretation of glucose data. In addition, hospitalization provides a structured environment in which therapeutic education can be delivered to patients and caregivers, and insulin regimens can be adjusted based on CGM profiles. This controlled setting may facilitate acceptance and early familiarization with CGM, thereby supporting continuation after discharge when clinically indicated, as suggested by our feasibility results.

The HYPOAGE study, which was conducted on 141 community-dwelling patients, found a median percentage of time between 54 and 70 mg/dL of 2.9% and a median time below 54 mg/dL of 0.7% [27]. In our study, the mean time below range (< 70 mg/dL) was 1.3%. Although glycemic metrics are not strictly comparable due to differences in thresholds and summary measures (median vs. mean), the overall hypoglycemic burden appears lower in our inpatient cohort. In a prospective pilot study involving 38 patients with an average age of 66.1 ± 8.6 years hospitalized in a general ward, CGM was shown to detect a higher number of hypoglycemic episodes than capillary blood glucose measurements [28]. Older adults using real-time CGM reported feeling safer thanks to alarms warning of impending hypoglycemia, which did not occur with fingerstick testing alone [19]. Various alarm options, especially hypoglycemia alerts, played a major role in caregivers’ acceptance of CGM when consulted. Furthermore, randomized trials among older adults demonstrate that compared to standard blood glucose monitoring, CGM use is associated with reductions in hypoglycemia. The GLADIS study encompassed 155 diabetic patients on CGM, dividing them into three groups: those not equipped with alarms, those with alarms, and those engaged in self-monitoring of blood glucose [29]. The authors concluded that the group with alarms spent significantly less time in hypoglycemia (0.6 h less per day) than the self-monitoring group, whereas such difference was not found in the group without alarms. Unfortunately, we could not collect the number of hypoglycemia alerts in our study. In our cohort, a mean of 1.2 hypoglycemic episodes per patient was observed. The lower proportion of hypoglycemia compared with the HYPOAGE cohort may be explained by methodological differences. In HYPOAGE, glucose data were collected using blinded CGM without alarms, whereas our study relied on real-time CGM with active hypoglycemia alerts. The availability of real-time glucose values and alarm notifications may have facilitated prompt clinical responses, including insulin dose adjustments and nutritional interventions, thereby possibly limiting the duration and recurrence of hypoglycemic events. In addition, the inpatient setting may have allowed closer metabolic monitoring than in the community-based HYPOAGE cohort.

There was no association between hypoglycemic events and patient-related variables such as age, autonomy, comorbidities, or diabetes parameters. Similar findings have been reported in older adults, where hypoglycemia risk was poorly predicted by traditional clinical factors, highlighting the complexity of glucose variability in this population [27, 30]. In our study, all hypoglycemic episodes were asymptomatic, reinforcing the well-known phenomenon of hypoglycemia unawareness in frail older patients. The absence of symptomatic hypoglycemia in our cohort is not unexpected in very old adults. Age-related impairment of autonomic counter-regulatory responses, long-standing diabetes, and cognitive vulnerability contribute to hypoglycemia unawareness in this population [31]. This observation supports systematic use of CGM in this setting, as conventional capillary glucose monitoring would have underdiagnosed these episodes.

One of the key findings of this study lies in the consistent correlations observed between insulin doses and CGM-derived glycemic metrics. Higher total and bolus insulin doses were associated with lower TIR and higher TAR, suggesting that, in this observational setting, intensification of insulin therapy does not necessarily translate into improved glycemic control in this frail geriatric population. These findings highlight the potential value of CGM in helping to identify suboptimal insulin strategies and supporting more individualized treatment adjustments in acute care settings. Patients who experienced hypoglycemia received lower weight-adjusted insulin doses. Although seemingly paradoxical, this finding may reflect increased insulin sensitivity in frail older adults, possibly related to reduced lean body mass, decreased oral intake, or renal impairment. In addition, insulin doses may have been down-titrated after the occurrence of hypoglycemic episodes during hospitalization, which could partly explain the inverse association observed. Regarding hyperglycemia, the positive correlations observed between TAR and insulin doses, particularly bolus doses, are somewhat counterintuitive. Higher insulin doses should theoretically reduce hyperglycemia; however, this pattern likely reflects reactive insulin adjustments in response to poor glycemic control, rather than representing the cause of elevated glucose levels. Similar associations have been described in inpatient cohorts, suggesting that intensified insulin therapy often reflects therapeutic inertia or overcorrection of postprandial hyperglycemia, without improvement in overall control [21, 32, 33]. These findings may also point to underlying insulin resistance, a phenomenon frequently observed in acutely ill older adults due to inflammation, infection, or glucocorticoid exposure.

Polypharmacy in older adults is commonly defined using numerical thresholds (often ≥ 5 medications), yet there is no universally accepted definition. Beyond the absolute number of prescribed drugs, the clinical appropriateness and necessity of each medication are crucial considerations. In geriatric medicine, the distinction between appropriate and inappropriate polypharmacy has been increasingly emphasized, as multiple comorbidities may legitimately require complex therapeutic regimens [34]. Therefore, medication burden should not be interpreted solely as a marker of vulnerability, but rather within a comprehensive clinical context that includes indication, benefit–risk balance, life expectancy, and patient-centered goals of care [35].

Taken together, our results illustrate the need for careful insulin management in older adults, in whom both under- and overtreatment can coexist. CGM could play a key role in guiding more dynamic and personalized insulin adjustments, helping clinicians to distinguish between true insulin resistance and therapeutic overcompensation.

Among patients who agreed to CGM initiation, acceptability was high, as most participants continued using the device throughout hospitalization. Discontinuation of CGM was infrequent and mainly related to clinical circumstances, such as in-hospital death or cessation of insulin therapy, rather than to device intolerance or refusal. This is consistent with recent real-world findings showing that patients discharged with CGM are generally willing to continue its use, provided that logistical and reimbursement barriers are addressed [36]. However, our observation that 61.5% of patients had clear indications for CGM according to national reimbursement criteria prior to hospitalization but that it had not been prescribed reflects well-documented barriers in the literature, including regulatory, logistical, and insurance-related obstacles [37]. A short stay in an acute geriatric unit may present a unique opportunity. During hospitalization, healthcare teams often have more time and resources to provide therapeutic education and support, facilitating initiation of CGM. Protocols describing discharge programs emphasize that structured patient education, clear discharge plans, and coordination between hospital and outpatient care are critical for maintaining CGM use post-discharge [38].

One of the strengths of our study was the inclusion of real-life patients, aged ≥ 75 years, with a high level of multimorbidity, admitted to an acute geriatric unit, a population often underrepresented in studies evaluating CGM. Importantly, CGM use in our study was not limited to short-term monitoring. With a median wearing time of 14 days, feasibility was assessed over a clinically meaningful period during which technical issues, alarm burden, or acceptability challenges are more likely to arise. This prolonged exposure strengthens the validity of our feasibility findings in an acute geriatric setting. Patient-reported outcomes were not collected, which limits our ability to assess perceived comfort, satisfaction, or quality-of-life benefits associated with CGM use in this acute geriatric population. This focus allowed us to specifically address feasibility and implementation aspects of CGM use in an acute geriatric setting.

One of the main limitations of our study was its pilot and observational design. The inclusion of 52 patients remains limited, and the absence of a control group precludes conclusions regarding the benefits of CGM in comparison to capillary blood glucose testing. The follow-up period was short, focusing on the period of hospitalization in an acute care unit. Moreover, we cannot exclude the possibility of pressure-induced sensor artefacts (“compression lows”), as hypoglycemic values were not systematically confirmed by capillary testing. However, this reflects real-world use of CGM in acute geriatric settings. In addition, the use of a scan-dependent CGM system may have influenced sensor activity rates; newer real-time CGM devices could potentially yield different feasibility results. Another limitation is the duration of CGM observation, which was constrained by the length of hospitalization. Although CGM was worn for several consecutive days in most patients, this period may not fully capture longer-term issues such as alarm fatigue, sensor-related complications, or sustained adherence over time. Therefore, our findings primarily reflect short-term feasibility during acute hospitalization rather than long-term sustainability.

Future studies should focus on treatment management tailored by CGM value and evaluate the involvement of the patient and self-management education during hospitalization. It would also be interesting to investigate the impact of CGM on patients’ quality of life during the hospital stay and after discharge. Several studies have suggested that CGM may reduce the stress of diabetes management [13]. The impact of this technology on caregivers and healthcare professionals, in the field of geriatrics, is another area for research [39]. Future research should explore its impact on therapeutic education, long-term adherence, and overall quality of life, particularly in post-hospitalization care. Expanding CGM access in geriatric populations could represent a significant step forward in personalized diabetes management, balancing safety, efficacy, and patient comfort. It should also be noted that the CGM system used in this study (FreeStyle Libre 2^®^) requires active scanning to obtain glucose values. Newer-generation CGM systems, such as FreeStyle Libre 3^®^, Dexcom G6/G7^®^, or Roche SmartGuide^®^, provide real-time continuous transmission without the need for scanning. These technological advances may further improve feasibility, reduce missing data related to scan frequency, and potentially enhance usability in older adults. Finally, recent advancements in technology have led to the development of novel devices that facilitate CGM. These devices employ machine learning algorithms to predict future blood glucose levels, offering a valuable tool for individuals with diabetes. This technology shows great promise for older patients with diabetes, for whom clinicians aim to prevent hypoglycemia and anticipate glycemic excursions rather than react to them [40, 41].

In conclusion, this study provides real-world evidence supporting the feasibility and short-term safety of CGM in acute geriatric wards, an underexplored yet crucial care setting. With a high continuation rate at discharge, CGM emerges as a valuable tool to optimize diabetes care in frail older adults, bridging inpatient and outpatient management.

## Supplementary Information

Supplementary Material 1: Supplementary Table 1: Comparison of baselines characteristics based on sensor activity. BMI: body mass index; CIRS-G: cumulative illness rate scale geriatric; ADL: activities of daily living; iADL: instrumental activities of daily living; eGFR: estimated glomerular filtration rate. Group 1: Patient with sensor activity ≥ 70% (analysis set). Group 2: Patients with sensor activity < 70%. Data are presented as median (IQR) or n (%). Patients were categorized post hoc according to CGM sensor activity (< 70% vs. ≥ 70%). Group comparisons were performed using Mann–Whitney U test for continuous variables and Fisher’s exact test or Chi² test for categorical variables. *Defined as eGFR ≤ 60 mL/min/1.73 m² according to MDRD. Supplementary Table 2: Study population and CGM data availability. Among patients with sensor activity ≥ 70% (*n* = 40): Discharged without CGM due to insulin discontinuation: 5. Death during hospitalization: 2. Sensor removal: 1. Discharged with CGM: 32. CGM-derived metrics were calculated only in patients with sensor activity ≥ 70% (*n* = 40). Supplementary Table 3: Comparison of insulin doses according to the occurrence of hypoglycemia in patients with ≥ 70% CGM sensor activity. Comparison of weight-adjusted daily insulin doses in patients with and without hypoglycemic events. Analyses were restricted to patients with ≥ 70% CGM sensor activity (*n* = 40). Hypoglycemia was defined as at least one CGM-recorded glucose value < 70 mg/dL during hospitalization. Data are expressed as median (interquartile range). Comparisons between groups were performed using the Mann–Whitney U test. Supplementary Table 4: Distribution of patients according to glycemic targets (*n* = 40). Values are expressed as mean ± standard deviation of the mean daily insulin dose (IU/kg/day). The green area represents the target zone, defined as the simultaneous achievement of all three international consensus targets: TIR > 50%, TAR < 50%, and TBR < 1%. TIR, Time in Range: percentage of time with sensor glucose between 70 and 180 mg/dL. TAR, Time Above Range: percentage of time with sensor glucose above 180 mg/dL (includes both TAR 180–250 mg/dL and TAR > 250 mg/dL). TBR, Time Below Range: percentage of time with sensor glucose below 70 mg/dL. IU, international units. Glycemic targets are based on the international consensus guidelines for CGM metrics (Battelino et al., Diabetes Care, 2019), adapted for older adults with type 2 diabetes: TIR > 50%, TAR < 50%, TBR < 1%. No statistical comparison was performed due to small subgroup sizes. Supplementary Fig. 1: Details of average insulin doses (basal and rapid) according to insulin regimen during hospitalization. Average dose of insulin per kilogram per day of slow and rapid analogue according to insulin regimen: basal (< 1 average bolus per day), basal and correction of rapid (between 1 and 2 average boluses per day) or basal-bolus regimen (> 2 average boluses per day). Supplementary Fig. 2: Number of hypoglycemic events per patient. Percentage of patients with or without hypoglycemic events (*n* = 52). The percentages presented reflect the distribution of patients according to the number of hypoglycemic events on continuous glucose monitoring (CGM) report, each value being calculated according to the total number of patients included in the study.

## Data Availability

The datasets used and/or analyzed during the current study are available from the corresponding author on reasonable request.

## References

[1] IDF Diabetes Atlas 10. th Edition. Disponible sur: https://diabetesatlas.org/data/. Cité 6 janv 2025.

[2] Diabetes Atlas - France. Diabetes Atlas. Disponible sur: https://diabetesatlas.org/data-by-location/country/france/. Cité 11 déc 2025.

[3] Diabète. Disponible sur: https://www.santepubliquefrance.fr/maladies-et-traumatismes/diabete. Cité 3 janv 2025.

[4] Umpierrez GE, Smiley D, Zisman A, Prieto LM, Palacio A, Ceron M, et al. Randomized study of basal-bolus insulin therapy in the inpatient management of patients with type 2 diabetes (RABBIT 2 trial). Diabetes Care sept. 2007;30(9):2181–6. 10.2337/dc07-0295. PubMed PMID: 17513708. 10.2337/dc07-0295 17513708

[5] Nakar S, Yitzhaki G, Rosenberg R, Vinker S. Transition to insulin in Type 2 diabetes: family physicians’ misconception of patients’ fears contributes to existing barriers. J Diabetes Complications. 2007;21(4):220–6. 02.004 PubMed PMID: 17616351. 17616351 10.1016/j.jdiacomp.2006.02.004

[6] Jeavons D, Hungin APS, Cornford CS. Patients with poorly controlled diabetes in primary care: healthcare clinicians’ beliefs and attitudes. Postgrad Med J mai. 2006;82(967):347–50. 10.1136/pgmj.2005.039545. PubMed PMID: 16679475; PubMed Central PMCID: PMC2563795. 10.1136/pgmj.2005.039545 PMC2563795 16679475

[7] Yk L, Cj N, Py L, Em K, Kl A, Wy L, et al. What are the barriers faced by patients using insulin? A qualitative study of Malaysian health care professionals’ views. Patient Prefer Adherence. 2013;7. 10.2147/PPA.S36791. PubMed PMID: 23378747. 10.2147/PPA.S36791 PMC3559078 23378747

[8] Arrêté du 8. juin 2023 portant modification des conditions d’inscription du système flash d’autosurveillance du glucose FREESTYLE LIBRE 2 de la société ABBOTT France inscrit au titre Ier de la liste des produits et prestations remboursables prévue à l’article L. 165-1 du code de la sécurité sociale - Légifrance. Disponible sur: https://www.legifrance.gouv.fr/jorf/id/JORFTEXT000047670319. Cité 9 févr 2025.

[9] CNEDIMTS-6266_FREESTYLE. LIBRE 2_20_octobre_2020_(6266)_avis.pdf. [cité 3 janv 2025]. Disponible sur: https://www.has-sante.fr/upload/docs/evamed/CNEDIMTS-6266_FREESTYLE%20LIBRE%202_20_octobre_2020_(6266)_avis.pdf.

[10] Cappon G, Vettoretti M, Sparacino G, Facchinetti A. Continuous Glucose Monitoring Sensors for Diabetes Management: A Review of Technologies and Applications. Diabetes Metab J août. 2019;43(4):383–97. 10.4093/dmj.2019.0121. PubMed PMID: 31441246; PubMed Central PMCID: PMC6712232. 10.4093/dmj.2019.0121 PMC6712232 31441246

[11] Cutruzzolà A, Irace C, Parise M, Fiorentino R, Pio Tripodi PF, Ungaro S, et al. Time spent in target range assessed by self-monitoring blood glucose associates with glycated hemoglobin in insulin treated patients with diabetes. Nutr Metabolism Cardiovasc Dis sept. 2020;30(10):1800–5. 10.1016/j.numecd.2020.06.009. 10.1016/j.numecd.2020.06.009 32669240

[12] Guerci B, Levrat-Guillen F, Vicaut E, De Pouvourville G, Detournay B, Emery C, et al. Reduced Acute Diabetes Events After FreeStyle Libre System Initiation in People 65 Years or Older with Type 2 Diabetes on Intensive Insulin Therapy in France. Diabetes Technol Ther juin. 2023;25(6):384–94. 10.1089/dia.2023.0034. PubMed PMID: 36944104. 10.1089/dia.2023.0034 36944104

[13] Prasad-Reddy L, Godina A, Chetty A, Isaacs D. Use of Continuous Glucose Monitoring in Older Adults: A Review of Benefits, Challenges and Future Directions. touchREV Endocrinol nov. 2022;18(2):116–21. 10.17925/EE.2022.18.2.116. PubMed PMID: 36694891; PubMed Central PMCID: PMC9835808. 10.17925/EE.2022.18.2.116 PMC9835808 36694891

[14] Battelino T, Danne T, Bergenstal RM, Amiel SA, Beck R, Biester T, et al. Clinical Targets for Continuous Glucose Monitoring Data Interpretation: Recommendations From the International Consensus on Time in Range. Diabetes Care août. 2019;42(8):1593–603. 10.2337/dci19-. 0028 PubMed PMID: 31177185; PubMed Central PMCID: PMC6973648. 10.2337/dci19-0028 PMC6973648 31177185

[15] Arrêté du 3 février 2025 portant. inscription du système flash d’autosurveillance du glucose interstitiel FREESTYLE LIBRE 2 PLUS de la société ABBOTT France au titre I de la liste des produits et prestations remboursables prévue à l’article L. 165-1 du code de la sécurité sociale - Légifrance. Disponible sur: https://www.legifrance.gouv.fr/jorf/id/JORFTEXT000051132535. Cité 19 févr 2025.

[16] American Diabetes Association Professional Practice Committee for Diabetes*. 13. Older adults: standards of care in diabetes—2026. Diabetes Care. 8 déc 2025;49(Supplement_1):S277–96. 10.2337/dc26-S013. 10.2337/dc26-S013 PMC12690186 41358888

[17] Battelino T, Alexander CM, Amiel SA, Arreaza-Rubin G, Beck RW, Bergenstal RM, et al. Continuous glucose monitoring and metrics for clinical trials: an international consensus statement. Lancet Diabetes Endocrinol janv. 2023;11(1):42–57. 10.1016/S2213-8587. (22)00319-9 PubMed PMID: 36493795. 10.1016/S2213-8587(22)00319-9 36493795

[18] INESSS. Système flash de surveillance du glucose (Freestyle libre). Rapport No. Disponible sur: https://www.inesss.qc.ca/fileadmin/doc/INESSS/Rapports/Technologies/INESSS_Avis_FreeStyle.pdf. Cité 6 janv 2025.

[19] Litchman ML, Allen NA. Real-Time Continuous Glucose Monitoring Facilitates Feelings of Safety in Older Adults With Type 1 Diabetes: A Qualitative Study. J Diabetes Sci Technol sept. 2017;11(5):988–95. doi:10.1177/1932296817702657 PubMed PMID: 28376647; PubMed Central PMCID: PMC5950993. 10.1177/1932296817702657 PMC5950993 28376647

[20] Barnard KD, Kropff J, Choudhary P, Neupane S, Bain SC, Kapitza C, et al. Acceptability of Implantable Continuous Glucose Monitoring Sensor. J Diabetes Sci Technol mai. 2018;12(3):634–8. doi:10.1177/1932296817735123 PubMed PMID: 28990436; PubMed Central PMCID: PMC6154244. 10.1177/1932296817735123 PMC6154244 28990436

[21] Leite SAO, Silva MP, Lavalle ACR, Bertogy MCV, Bastos M, Kuklik SCV, et al. Use of continuous glucose monitoring in insulin-treated older adults with type 2 diabetes. Diabetol Metabolic Syndrome 23 nov. 2023;15(1):240. 10.1186/s13098-023-01225-4. 10.1186/s13098-023-01225-4 PMC10666454 37993898

[22] Alfadli SF, Alotaibi YS, Aqdi MJ, Almozan LA, Alzubaidi ZB, Altemani HA, et al. Effectiveness of continuous glucose monitoring systems on glycemic control in adults with type 1 diabetes: A systematic review and meta-analysis. Metabol Open sept. 2025;27:100382. 10.1016/j.metop.2025.100382. PubMed PMID: 40791933; PubMed Central PMCID: PMC12337206. 10.1016/j.metop.2025.100382 PMC12337206 40791933

[23] Smith MB, Albanese-O’Neill A, Macieira TGR, Yao Y, Abbatematteo JM, Lyon D, et al. Human Factors Associated with Continuous Glucose Monitor Use in Patients with Diabetes: A Systematic Review. Diabetes Technol Ther oct. 2019;21(10):589–601. 10.1089/dia.2019.0136. PubMed PMID: 31335196. 10.1089/dia.2019.0136 31335196

[24] Galindo RJ, Aleppo G, Klonoff DC, Spanakis EK, Agarwal S, Vellanki P, et al. Implementation of Continuous Glucose Monitoring in the Hospital: Emergent Considerations for Remote Glucose Monitoring During the COVID-19 Pandemic. J Diabetes Sci Technol 14 juin. 2020;14(4):822–32. /1932296820932903 PubMed PMID: 32536205; PubMed Central PMCID: PMC7673156. 10.1177/1932296820932903 PMC7673156 32536205

[25] Maltese G, McAuley SA, Trawley S, Sinclair AJ. Ageing well with diabetes: the role of technology. Diabetologia oct. 2024;67(10):2085–102. 10.1007/s00125-024-06240-2. PubMed PMID: 39138689; PubMed Central PMCID: PMC11446974. 10.1007/s00125-024-06240-2 PMC11446974 39138689

[26] Purnamayanti NKD, Wicaksana AL. Digital Health Services among Patients with Diabetes during the COVID-19 Pandemic: A Scoping Review. Indian J Endocrinol Metab. 2021;25(2):86–92. 10.4103/ijem.ijem_153_21. PubMed PMID: 34660235; PubMed Central PMCID: PMC8477741. 34660235 10.4103/ijem.ijem_153_21 PMC8477741

[27] Boureau AS, Guyomarch B, Gourdy P, Allix I, Annweiler C, Cervantes N, et al. Nocturnal hypoglycemia is underdiagnosed in older people with insulin-treated type 2 diabetes: The HYPOAGE observational study. J Am Geriatr Soc juill. 2023;71(7):2107–19. 10.1111/jgs.18341. PubMed PMID: 36965179. 10.1111/jgs.18341 36965179

[28] Gómez AM, Umpierrez GE, Muñoz OM, Herrera F, Rubio C, Aschner P, et al. Continuous Glucose Monitoring Versus Capillary Point-of-Care Testing for Inpatient Glycemic Control in Type 2 Diabetes Patients Hospitalized in the General Ward and Treated With a Basal Bolus Insulin Regimen. J Diabetes Sci Technol 31 août. 2015;10(2):325–9. doi:10.1177/1932296815602905 PubMed PMID: 26330394; PubMed Central PMCID: PMC4773955. 10.1177/1932296815602905 PMC4773955 26330394

[29] New JP, Ajjan R, Pfeiffer AFH, Freckmann G. Continuous glucose monitoring in people with diabetes: the randomized controlled Glucose Level Awareness in Diabetes Study (GLADIS). Diabet Med mai. 2015;32(5):609–17. 10.1111/dme.12713. PubMed PMID: 25661981. 10.1111/dme.12713 25661981

[30] Christiaens A, Boureau AS, Guyomarch B, de Decker L, Boland B, Hadjadj S, et al. Diabetes Overtreatment and Hypoglycemia in Older Patients With Type 2 Diabetes on Insulin Therapy: Insights From the HYPOAGE Cohort Study. Diabetes Care 1 janv. 2025;48(1):61–6. 10.2337/dc24-. 1058 PubMed PMID: 39172937. 10.2337/dc24-1058 39172937

[31] Norman K, Stobäus N, Pirlich M, Bosy-Westphal A. Bioelectrical phase angle and impedance vector analysis–clinical relevance and applicability of impedance parameters. Clin Nutr déc. 2012;31(6):854–61. 10.1016/j.clnu.2012.05.008. PubMed PMID: 22698802. 10.1016/j.clnu.2012.05.008 22698802

[32] Huang Y, Heng C, Wei J, Jing X, Wang X, Zhao G, et al. Influencing factors of glycemic variability in hospitalized type 2 diabetes patients with insulin therapy: A Strobe-compliant article. Med (Baltimore) sept. 2017;96(36):e8021. 10.1097/MD.0000000000008021. PubMed PMID: 28885369; PubMed Central PMCID: PMC6392839. 10.1097/MD.0000000000008021 PMC6392839 28885369

[33] Price C, Callahan KE, Aloi JA, Usoh CO. Continuous Glucose Monitoring in Older Adults: What We Know and What We Have Yet to Learn. J Diabetes Sci Technol 7 mars. 2024;18(3):577–83. doi:10.1177/19322968241234651 PubMed PMID: 38454549; PubMed Central PMCID: PMC11089865. 10.1177/19322968241234651 PMC11089865 38454549

[34] Nicholson K, Liu W, Fitzpatrick D, Hardacre KA, Roberts S, Salerno J, et al. Prevalence of multimorbidity and polypharmacy among adults and older adults: a systematic review. Lancet Healthy Longev avr. 2024;5(4):e287–96. 10.1016/S2666-7568. (24)00007-2 PubMed PMID: 38452787. 10.1016/S2666-7568(24)00007-2 38452787

[35] Pazan F, Wehling M. Polypharmacy in older adults: a narrative review of definitions, epidemiology and consequences. Eur Geriatr Med juin. 2021;12(3):443–52. 10.1007/s41999-021-00479-3. PubMed PMID: 33694123; PubMed Central PMCID: PMC8149355. 10.1007/s41999-021-00479-3 PMC8149355 33694123

[36] Wilson A, Morrison D, Sainsbury C, Jones G. Narrative Review: Continuous Glucose Monitoring (CGM) in Older Adults with Diabetes. Diabetes Ther juin. 2025;16(6):1139–54. 10.1007/s13300-025-01720-z. PubMed PMID: 40238078; PubMed Central PMCID: PMC12085541. 10.1007/s13300-025-01720-z PMC12085541 40238078

[37] Tian T, Aaron RE, Seley JJ, Longo R, Nayberg I, Umpierrez GE, et al. Use of Continuous Glucose Monitors Upon Hospital Discharge of People With Diabetes: Promise, Barriers, and Opportunity. J Diabetes Sci Technol janv. 2024;18(1):207–14. 19322968231200847 PubMed PMID: 37784246; PubMed Central PMCID: PMC10899827. 10.1177/19322968231200847 PMC10899827 37784246

[38] Kumar N, Knight AM, Demidowich AP, Stanback CF, Bashura H, Hussain Q et al. Implementing a Continuous Glucose Monitoring Hospital Discharge Program: Strategies and Best Practices. J Diabetes Sci Technol. 2025;19322968251370754. 10.1177/19322968251370754. PubMed PMID: 40958410. 10.1177/19322968251370754 PMC13044410 40958410

[39] Lawton J, Blackburn M, Allen J, Campbell F, Elleri D, Leelarathna L, et al. Patients’ and caregivers’ experiences of using continuous glucose monitoring to support diabetes self-management: qualitative study. BMC Endocr Disord 20 févr. 2018;18(1):12. 10.1186/s12902-018-0239-1. PubMed PMID: 29458348; PubMed Central PMCID: PMC5819241. 10.1186/s12902-018-0239-1 PMC5819241 29458348

[40] Kahkoska AR, Shah KS, Kosorok MR, Miller KM, Rickels M, Weinstock RS, et al. Estimation of a Machine Learning-Based Decision Rule to Reduce Hypoglycemia Among Older Adults With Type 1 Diabetes: A Post Hoc Analysis of Continuous Glucose Monitoring in the WISDM Study. J Diabetes Sci Technol sept. 2024;18(5):1079–86. doi:10.1177/19322968221149040 PubMed PMID: 36629330; PubMed Central PMCID: PMC11418529. 10.1177/19322968221149040 PMC11418529 36629330

[41] Glatzer T, Ringemann C, Militz D, Mueller-Hoffmann W. Concept and Implementation of a Novel Continuous Glucose Monitoring Solution With Glucose Predictions on Board. J Diabetes Sci Technol sept. 2024;18(5):1004–8. doi:10.1177/19322968241269927 PubMed PMID: 39158990; PubMed Central PMCID: PMC11418471. 10.1177/19322968241269927 PMC11418471 39158990

