# Supplementary material for: Feasibility of continuous glucose monitoring in an acute geriatric unit – the GLYCOGER study
Source: BMC Geriatr. 2026 Mar 17;26:578. doi: 10.1186/s12877-026-07333-w (PMC13107580; doi:10.1186/s12877-026-07333-w)
Supplement: Supplementary file 1 — Supplementary Material 1: Supplementary Table 1: Comparison of baselines characteristics based on sensor activity. BMI: body mass index; CIRS-G: cumulative illness rate scale geriatric; ADL: activities of daily living; iADL: instrumental activities of daily living; eGFR: estimated glomerular filtration rate. Group 1: Patient with sensor activity ≥ 70% (analysis set). Group 2: Patients with sensor activity < 70%. Data are presented as median (IQR) or n (%). Patients were categorized post hoc according to CGM sensor activity (< 70% vs. ≥ 70%). Group comparisons were performed using Mann–Whitney U test for continuous variables and Fisher’s exact test or Chi² test for categorical variables. *Defined as eGFR ≤ 60 mL/min/1.73 m² according to MDRD. Supplementary Table 2: Study population and CGM data availability. Among patients with sensor activity ≥ 70% (n = 40): Discharged without CGM due to insulin discontinuation: 5. Death during hospitalization: 2. Sensor removal: 1. Discharged with CGM: 32. CGM-derived metrics were calculated only in patients with sensor activity ≥ 70% (n = 40). Supplementary Table 3: Comparison of insulin doses according to the occurrence of hypoglycemia in patients with ≥ 70% CGM sensor activity. Comparison of weight-adjusted daily insulin doses in patients with and without hypoglycemic events. Analyses were restricted to patients with ≥ 70% CGM sensor activity (n = 40). Hypoglycemia was defined as at least one CGM-recorded glucose value < 70 mg/dL during hospitalization. Data are expressed as median (interquartile range). Comparisons between groups were performed using the Mann–Whitney U test. Supplementary Table 4: Distribution of patients according to glycemic targets (n = 40). Values are expressed as mean ± standard deviation of the mean daily insulin dose (IU/kg/day). The green area represents the target zone, defined as the simultaneous achievement of all three international consensus targets: TIR > 50%, TAR < 50%, and TBR < 1%. TIR, Time in [file 12877_2026_7333_MOESM1_ESM.docx]

# Supplementary Table 1: Comparison of baselines characteristics based on sensor activity

|  | Group 1 (n=40, 76.9%)  Median (IQR) or n (%) | Group 2 (n=12, 23.1%)  Median (IQR) or n (%) | | p-value |
| --- | --- | --- | --- | --- |
| Patients’ characteristics | | | | |
| Age, years | 86.0 (81.0-89.3) | 88.0 (81.8-89.3) | | 0.91 |
| Gender - Female | 17 (42.5) | 9 (75.0) | | 0.10 |
| BMI (kg/m²) | 28.3 (23.0- 31.9) | 31.3 (24.9-37.2) | | 0.25 |
| CIRS-G | 12.0 (10.0-15.0) | 16.0 (14.3- 17.5) | | 0.08 |
| ADL (0-6) | 4.8 (3.5-5.5) | 5.5 (3.8-6.0) | | 0.39 |
| iADL (0-8) | 4.0 (1.0-5.0) | 3.0 (1.8-4.8) | | 0.94 |
| Living situation prior to admission |  |  | | 0.44 |
| Home | 35 (87.5) | 12 (100.0) | |  |
| Nursing home | 4 (10.0) | 0 (0.0) | |  |
| Residential care facility | 1 (2.5) | 0 (0.0) | |  |
| Number of drugs per day at admission | 11.0 (8.0-12.0) | 10.0 (8.5-12.3) | | 0.89 |
| Biological values at admission | | | | |
| Fasting glucose (mg/dL) | 168.0 (122.0-211.50) | 151.5 (101.8-188.5) | | 0.44 |
| Glycated hemoglobin (mmol/L) | 10.0 (9.0-10.9) | 10.3 (9.0-12.2) | | 0.72 |
| Glycated hemoglobin (%) | 7.9 (7.3-8.5) | 8.1 (7.3-9.3) | | 0.72 |
| Creatinine (μmol/L) | 100.0 (77.0- 151.3) | 101.5 (86.3-138.5) | | 0.69 |
| eGFR (mL/min/1,73m²) | 50.0 (36.0- 71.5) | 55.0 (37.7- 73.8) | | 0.91 |
| History of diabetes | | |  |  |
| Type of diabetes |  |  | |  |
| Type 2 diabetes | 38 (95.0) | 12 (100.0) | | 1.000 |
| Secondary | 2 (5.0) | 0 (0.0) | |  |
| Diabetes Duration (years) |  |  | |  |
| <10 | 18 (45.0) | 1 (9.1) | | 0.067 |
| ≥10 | 22 (55.0) | 11 (90.9) | |  |
| Antidiabetic drugs prior to hospital admission |  |  | | 0.64 |
| Oral antidiabetic only | 13 (32.5) | 4 (33.3) | |  |
| Insulin only | 11 (27.5) | 5 (41.7) | |  |
| Oral antidiabetic and insulin | 13 (32.5) | 3 (25.0) | |  |
| None | 3 (7.5) | 0 (0.0) | |  |
| Diabetes complications |  |  | |  |
| Any microangiopathic complication | 31 (77.5) | 10 (83.3) | | 0.98 |
| Retinopathy | 6 (15.0) | 2 (16.7) | | 1.00 |
| Nephropathy* | 26 (65.0) | 7 (58.3) | | 0.66 |
| Neuropathy | 6 (15.0) | 5 (41.7) | | 0.11 |
| Any macroangiopathic complication | 26 (65.0) | 10 (83.3) | | 0.40 |
| Stroke | 14 (35.0) | 5 (41.7) | | 0.94 |
| Myocardial infarction | 12 (30.0) | 5 (41.7) | | 0.68 |
| Peripheral arterial disease | 8 (20.0) | 3 (25.0) | | 1.00 |

BMI: body mass index; CIRS-G: cumulative illness rate scale geriatric; ADL: activities of daily living; iADL: instrumental activities of daily living; eGFR: estimated glomerular filtration rate

Group 1: Patient with sensor activity ≥ 70% (analysis set)

Group 2: Patients with sensor activity < 70%

Data are presented as median (IQR) or n (%). Patients were categorized post hoc according to CGM sensor activity (<70% vs ≥70%). Group comparisons were performed using Mann–Whitney U test for continuous variables and Fisher’s exact test or Chi² test for categorical variables.

*Defined as eGFR≤ 60 mL/min/1.73m² according to MDRD

# Supplementary table 2: Study population and CGM data availability

| Subgroup | n (%) |
| --- | --- |
| Included patients | 52 (100) |
| Patients with ≥ 7 days of CGM | 52 (100) |
| Patient with sensor activity ≥ 70% (analysis set) | 40 (77) |
| Patients with sensor activity < 70% | 12 (33) |

Among patients with sensor activity ≥70% (n = 40):

Discharged without CGM due to insulin discontinuation: 5

Death during hospitalization: 2

Sensor removal: 1

Discharged with CGM: 32

CGM-derived metrics were calculated only in patients with sensor activity ≥70% (n=40).

Supplementary Table 3: Comparison of insulin doses according to the occurrence of hypoglycemia in patients with ≥70% CGM sensor activity

|  | Hypoglycemia (n=16) | No hypoglycemia (n=24) | p-value |
| --- | --- | --- | --- |
| Total daily insulin (IU/kg/day) | 0.35 (0.19-0.48) | 0.40 (0.28-0.51) | 0.372 |
| Basal insulin (IU/kg/day) | 0.20 (0.13-0.23) | 0.24 (0.14-0.27) | 0.301 |
| Bolus insulin (IU/kg/day) | 0.14 (0.06-0.25) | 0.18 (0.10-0.22) | 0.681 |

Comparison of weight-adjusted daily insulin doses in patients with and without hypoglycemic events.

Analyses were restricted to patients with ≥70% CGM sensor activity (n = 40). Hypoglycemia was defined as at least one CGM-recorded glucose value <70 mg/dL during hospitalization.

Data are expressed as median (interquartile range). Comparisons between groups were performed using the Mann–Whitney U test.

Supplementary Table 4: Distribution of patients according to glycemic targets (n=40)

|  | TBR <1% | TBR ≥ 1% |
| --- | --- | --- |
| TIR > 50% + TAR < 50% (n=30) | n=15  Total: 0.40±0.14  Basal: 0.21±0.08  Bolus: 0.19±0.09 | n=15  Total: 0.23±0.13  Basal: 0.14±0.07  Bolus: 0.08±0.07 |
| TIR <50% + TAR > 50% (n=10) | n=8  Total: 0.51±0.24  Basal: 0.29±0.18  Bolus: 0.21±0.08 | n=2  Total: 0.58±0.10  Basal: 0.27±0.03  Bolus: 0.30±0.07 |

Values are expressed as mean ± standard deviation of the mean daily insulin dose (IU/kg/day).

The green area represents the target zone, defined as the simultaneous achievement of all three international consensus targets: TIR >50%, TAR <50%, and TBR <1%.

TIR, Time in Range: percentage of time with sensor glucose between 70 and 180 mg/dL. TAR, Time Above Range: percentage of time with sensor glucose above 180 mg/dL (includes both TAR 180–250 mg/dL and TAR >250 mg/dL). TBR, Time Below Range: percentage of time with sensor glucose below 70 mg/dL. IU, international units.

Glycemic targets are based on the international consensus guidelines for CGM metrics (Battelino et al., Diabetes Care, 2019), adapted for older adults with type 2 diabetes: TIR >50%, TAR <50%, TBR <1%.

No statistical comparison was performed due to small subgroup sizes.

Supplementary Figure 1: Details of average insulin doses (basal and rapid) according to insulin regimen during hospitalization

Average dose of insulin per kilogram per day of slow and rapid analogue according to insulin regimen: basal (< 1 average bolus per day), basal and correction of rapid (between 1 and 2 average boluses per day) or basal-bolus regimen (>2 average boluses per day).

Supplementary Figure 2: Number of hypoglycemic events per patient


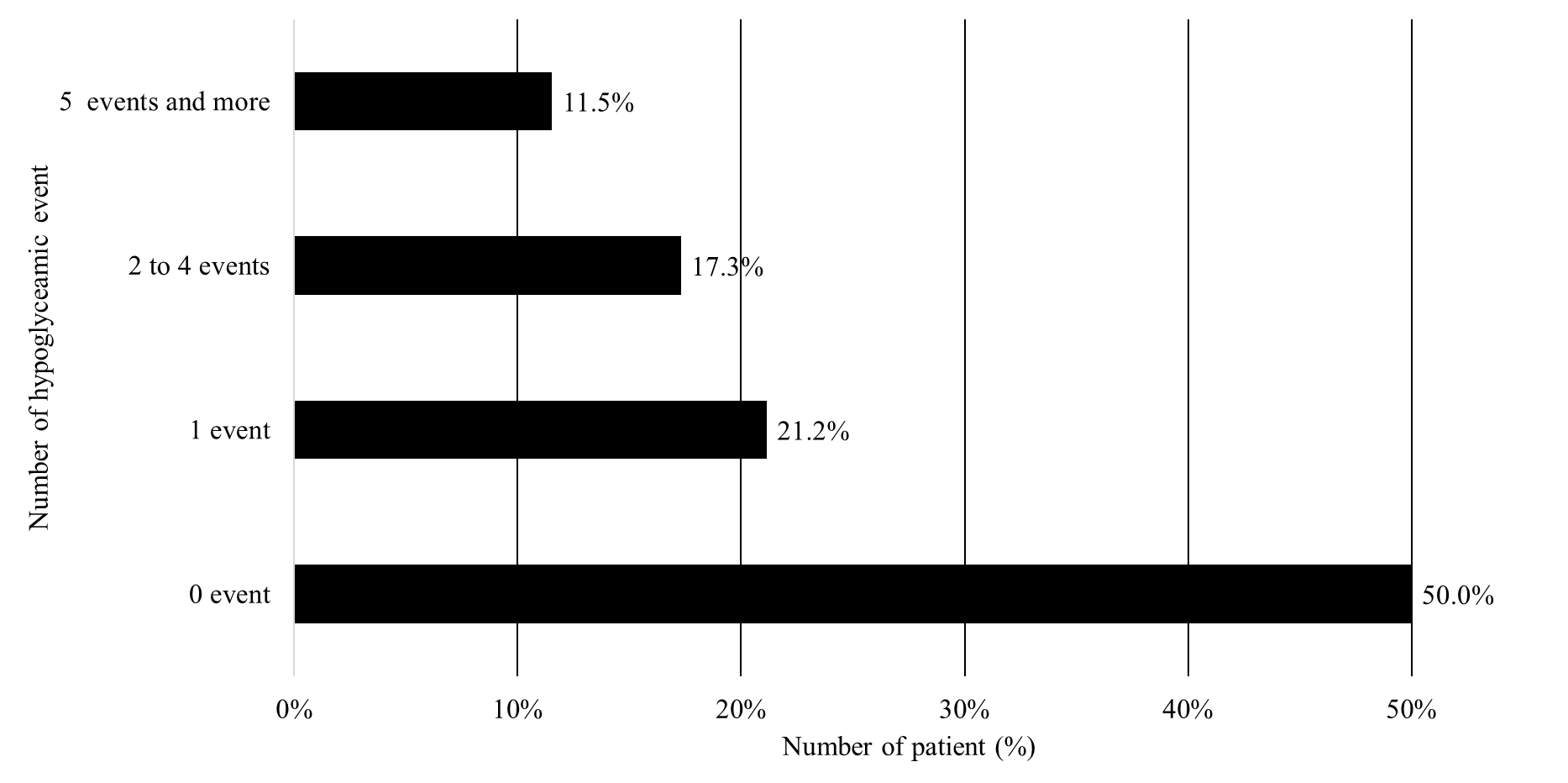


Percentage of patients with or without hypoglycemic events (n=52). The percentages presented reflect the distribution of patients according to the number of hypoglycemic events on continuous glucose monitoring (CGM) report, each value being calculated according to the total number of patients included in the study.
